# *Theileria* infection in bullfighting cattle in Thailand

**DOI:** 10.14202/vetworld.2022.2917-2921

**Published:** 2022-12-23

**Authors:** Pirayu Rakwong, Narissara Keawchana, Ruttayaporn Ngasaman, Ketsarin Kamyingkird

**Affiliations:** 1 Faculty of Veterinary Science, Prince of Songkla University, Hat Yai, Songkhla, Thailand;; 2 Department of Parasitology, Faculty of Veterinary Medicine, Kasetsart University, Ladyao, Bangkok, Thailand

**Keywords:** bullfighting cattle, hematological alteration, molecular identification, Thailand, *Theileria*

## Abstract

**Background and Aim:**

An apicomplexan protozoan parasite, namely, *Theileria*, primarily causes theileriosis in cattle worldwide. The virulence of the disease has been neglected because of it’s low pathogenicity. However, the disease can have a substantial effect, depending on the virulence of the species, low host immunity, and coinfection. In Thailand, the molecular detection of *Theileria* infection in bullfighting cattle and its hematological alterations have not been reported. Thus, this study aimed to identify *Theileria* species in bullfighting cattle in Thailand.

**Materials and Methods:**

Blood samples were collected from bullfighting cattle presented at the Prince of Songkla University Animal Hospital and were determined on the basis of hematological evaluation and DNA extraction. Molecular detection using the *18s rRNA* and merozoite surface antigen genes was conducted for *Theileria* spp. and *Theileria* *orientalis*, respectively. In addition, bidirectional sequencing of the positive samples was performed. Hematological alterations between *Theileria* infected and uninfected groups were statistically evaluated.

**Results:**

The levels of *Theileria* spp. and *T. orientalis* infection in bullfighting cattle were 44.62% (58/130) and 41.54% (54/130), respectively. *Theileria orientalis*, *Theileria sinensis*, and *Theileria* spp. infections were identified in bullfighting cattle samples. Hematological evaluation indicated that the red blood cell (RBC) level was significantly lower in *Theileria-*infected cattle.

**Conclusion:**

This study was the first to use molecular techniques in the identification of *Theileria* infection in bullfighting cattle in Thailand, with nearly one-half of the study population infected. *Theileria* infection in bullfighting cattle altered the RBC level, resulting in anemia. Therefore, tick control measures should be promoted.

## Introduction

*Theileria* is a protozoan parasite belonging to the phylum *Apicomplexa*, order Conoidasida, and genus *Theileria*, which is known as piroplasm. It is a tick-borne hemoparasite that causes theileriosis, which has been reported worldwide [1]. Bovine theileriosis is due to many species, including *Theileria* *parva*, *Theileria* *annulata*, *Theileria* *orientalis*, and *Theileria* *sinensis* [1]. In Thailand, benign *Theileria* species have been reported, including *Theileria* *buffeli*, *Theileria* *sergenti*, *Theileria* spp. type Thai [2], type Thung Song [3], type C [4, 5], and type B1 [5]. *Theileria* *orientalis* types 1, 3, 4, 5, 6, 7, N2, and N3 were reported in cattle and buffalo using the merozoite surface antigen (*MPSP*) gene [6, 7]. The clinical signs in infected animals included fever, anemia, jaundice, superficial lymph node enlargement, lethargy, lack of appetite, and exercise intolerance [8]. Theileriosis may cause a high ratio of mortality in pregnant cows and small ruminants [9]. Pregnant cows may abort or develop anemia during late gestation to 2–4 months after calving [10], whereas dairy cows may show a decrease in milk production [11]. The loss of draft and reduced strength of livestock can lead to large economic losses.

Bullfighting cattle are raised as pets and used for competition, particularly in South Thailand. Generally, the identification of *Theileria* in blood collected from bullfighting cattle results from regular health examinations before or after fighting competitions. Microscopic examination for the identification of blood parasites is conducted using a thin blood smear stained with Giemsa or modified Wright Giemsa. However, microscopic examination can only report the genus of piroplasms; hence, it is a low-sensitivity method. Previously, the infection level of *Theileria* in bullfighting cattle was identified by microscopic examination at 38.20% prevalence [12]. However, *Theileria* infection in bullfighting cattle in Thailand lacks species identification. Therefore, this study aimed to identify *Theileria* infection using molecular methods and to confirm the species of *Theileria* in bullfighting cattle in South Thailand.

## Materials and Methods

### Ethical approval

This study was approved by the Kasetsart University Institute of Animal Use and Care Committee, Bangkok, Thailand (approval no. ACKU01360).

### Study period and location

Sample collection was conducted from August 2020 to March 2021. Molecular identification, analysis, and interpretation were conducted from March to June 2021 at Faculty of Veterinary Science, Prince of Songkla University. All animals included in this study were raised in South Thailand.

### Sample collection

The bulls were brought to the Prince of Songkla Animal Hospital for health monitoring after bullfighting competitions from August 2020 to March 2021 for a health check-up. Licensed veterinarians collected blood samples through jugular venipuncture (approximately 10 mL from each animal). Approximately 3 mL of the whole-blood sample was placed in an ethylenediaminetetraacetic acid blood collection tube for hematological evaluation and DNA extraction. Then, the remaining blood sample was placed in a dry tube to harvest serum for blood biochemical analysis.

### Hematological and blood biochemical profile evaluation

Hematological and blood biochemical profiles were measured at the Hematology and Biochemistry Laboratory Unit, Veterinary Diagnostic Center, Faculty of Veterinary Science, Prince of Songkla University, Thailand. A complete blood count was analyzed using a BC-2800 Vet Auto Hematology Analyzer (Mindray^®^, China). Thin blood smears were prepared, air-dried, and fixed in absolute methanol (J.T. Baker^®^, USA). Afterward, the prepared thin smears were stained with 10% Giemsa stain (Merck^®^, Germany) in a buffer (pH 7.4) for about 12 min and then rinsed with tap water and allowed to dry. A differential count of white blood cells (WBCs) as well as the presence and identification of blood parasites were determined under a light microscope (Nikon Eclipse E200, Japan). The following blood biochemical parameters were measured: Plasma protein (PP), aspartate aminotransferase (AST), blood urea nitrogen (BUN), creatinine (Cre), creatine kinase (CK), and gamma-glutamyltransferase (GGT). Blood biochemical analysis was performed using a BS-120 Chemistry Analyzer (Mindray^®^, China).

### Molecular identification of *Theileria*

#### DNA extraction

Two hundred microliters of whole blood was used for DNA extraction with a DNA extraction kit (Geneaid Biotech Ltd., Taiwan) as recommended by the manufacturer. DNA concentration was measured using a UV–visible spectrophotometer (NanoDrop™, Thermo Fisher Scientific, USA) and stored at −20°C before molecular assay.

#### Identification of Theileria spp. using the 18s rRNA gene

Identification of *Theileria* spp. *18s rRNA* gene was conducted as described previously by Cao *et al*. [13]. In brief, 12 μL of polymerase chain reaction (PCR) reaction was prepared, containing 5 μL of Taq polymerase master mix buffer (KAPA^®^, Japan), 0.5 μL of each primer (100 μM), 4 μL of distilled water (DW), and 2 μL of DNA template. The thermocycler conditions were as follows: Initial denaturation at 95°C for 5 min, 35 cycles of denaturation at 94°C for 1 min, annealing at 55°C for 1 min, extension at 72°C for 1 min, and final extension at 72°C for 7 min (BioRad, USA). The negative and *Theileria-*positive controls were used as a DW sample and a microscopic positive *Theileria* DNA sample, respectively. The PCR products were migrated in an electrophoresis chamber using 1.5% of agarose gel. The positive PCR products were dissected, purified using a gel extraction kit (Geneaid Biotech Ltd.), and sent for DNA sequencing.

#### Identification of T. orientalis using the MPSP gene

The identification of *T. orientalis* *MPSP* gene was conducted as described previously by Ota *et al*. [14]. The PCR master mix was prepared as previously described. The thermocycler conditions were as follows: Initial denaturation at 95°C for 5 min, 35 cycles of denaturation at 94°C for 45 s, annealing at 58°C for 1 min, extension at 72°C for 1 min, and final extension at 72°C for 7 min (BioRad). The negative and *T. orientalis*-positive controls were a DW sample and a DNA sample extracted from *Theileria* spp. microscopic-positive cattle, respectively. The positive PCR products were used as described previously by Ota *et al*. [14].

#### Gel electrophoresis and DNA sequencing

The PCR products were migrated in 1.5% of agarose gel (1^st^ Base, Axil Scientific Pte. Ltd., Singapore) and visualized under ImageQuant™ LAS500 (GE Healthcare Life Science, USA). The *18s rRNA* and *MPSP* genes revealed PCR products of 778 and 776 bp, respectively. The PCR products were dissected and purified using a gel extraction kit (Geneaid Biotech Ltd.). Bidirectional sequencing was performed at ATGC Co. Ltd., Thailand. Nucleotide sequences were analyzed using UNIPRO GENE Version 41.0 software (http://ugene.net/), and Basic Local Alignment Search Tool was conducted through the U.S. National Library of Medicine National Center for Biotechnology Information website.

### Statistical analysis

Statistical analysis, including the percentage of *Theileria* spp. infection and the characteristics of the study samples, was performed using Microsoft Excel (Microsoft Corp., Washington, USA). Hematological alteration and blood biochemical parameters of samples from *Theileria* spp. infected and uninfected bulls were analyzed using an online t-test calculator (https://www.socscistatistics.com/test/t-test, accessed July 30, 2022).

## Results

### Characteristics of the study population

This study included 130 bullfighting cattle from five different provinces (Nakhon Si Thammarat, Phatthalung, Satun, Songkhla, and Trang). The cattle were all males with an age range of 2–4 years. Weakness and gasping were also observed in some bulls.

### Molecular identification of *Theileria spp*. and *T. orientalis* in bullfighting cattle

The level of *Theileria* spp. infection in cattle was 44.62% (58/130) based on the *18s rRNA* gene target. Sequencing of 11 PCR products from *18s rRNA* gene-positive samples confirmed 6/11 (54.54%) of *Theileria* spp., 4/11 (36.36%) of *T. sinensis*, and 1/11 (9.09%) of *Babesia bovis*. Sequencing analysis showed 99.73%–100% identity to the data published in GenBank.

The level of *T. orientalis* infection in cattle was 41.54% (54/130) using the *MPSP* gene target. Sequencing of 7 PCR products from *MPSP* gene-positive samples confirmed the presence of *T. orientalis* with 98.98%–99.07% identity to data published in GenBank. In this study, *T. orientalis* was the predominant species infecting the cattle (41.54%; 54/130; Table-1).

**Table-1:** Molecular identification of *Theileria* spp. and *T. orientalis* infection in bullfighting cattle, Thailand.

Target gene	Total sample	Positive sample	Percentage
*18s rRNA Theileria* spp.	130	58	44.62
*MPSP T. orientalis*		54	41.54

*T. orientalis*: *Theileria orientalis*

### Hematological alterations

Given the small number of variables, t-test analysis was used to compare the average hematological and blood biochemical values between *Theileria* spp. infected and uninfected groups. The comparison of differential counts between *Theileria* spp. infected and uninfected groups of bullfighting cattle showed that the red blood cell (RBC) level of *Theileria* spp. infected group was significantly lower than that of *Theileria* spp. uninfected group (p < 0.05). The WBC level and percentage of segment–neutrophils (N), lymphocytes, and eosinophils of *Theileria* spp. infected group were slightly lower than those of the *Theileria* spp. uninfected group. However, the percentage of lymphocytes, mean corpuscular volume, red cell distribution width, and platelet of *Theileria* spp. infected group was slightly higher than that of the uninfected group. The average values of blood biochemical parameters of *Theileria* spp. infected group, including PP, AST, BUN, Cre, CK, and GGT, were slightly higher than those of the uninfected group. However, no significant differences were observed (p > 0.05, Table-2). Furthermore, the AST, Cre, and CK levels were higher than the reference range [15].

**Table-2:** Hematological and blood biochemical parameters for *Theileria* spp. between infected and uninfected bullfighting cattle, Thailand.

Parameters	*Theileria*-infected bullfighting cattle	Min-Max value	*Theileria*-uninfected bullfighting cattle	Min-Max value	p-value	Reference range^a^
Differential count						
RBC (cells×10^6^)	6.91	5.09–9.59	7.26	4.61–9.46	0.0386*	5.0–7.2
WBC (cells×10^3^)	8.33	3.1–18	9.21	0.8–88	0.508	5.9–14.0
Segment-N (%)	47.43	3.0–78.0	49.29	19.0–81	0.375	1.8–7.2
Band-N (%)	0.12	0–2	0.04	0–2	0.221	0
Lymphocyte (%)	35.34	15–64	32.88	8–70	0.19	1.7–7.5
Monocyte (%)	3.84	1–10	4.49	0–14	0.171	0–0.9
Eosinophil (%)	12.69	1–41	13.32	0–39	0.634	0–1.3
Basophil (%)	0.00	0	0.00	0	0	0–0.3
Hematological parameters						
Hb (g/dl)	11.95	8.3–16.7	12.31	1.5–16.1	0.262	8.7–12.4
PCV (%)	35.90	23.0–47.0	37.06	3.0–49.0	0.227	25–33
MCV (fL)	56.92	47.4–71.2	56.42	36.8–67.9	0.522	38–51
MCH (pg)	17.32	14.2–21.1	17.16	11.0–20.0	0.464	14–19
MCHC (g/dl)	30.46	24.6–33.4	30.51	28.4–33.8	0.787	34–38
RDW (%)	16.27	14.8–18.3	16.20	14.3–21.7	0.661	15.0–19.4
PLT (cells×10^3^)	314.07	44–771	311.61	22.0–510	0.899	252–724
Blood biochemical parameters						
PP (g/dL)	7.59	6.0–8.7	7.64	5–8.6	0.605	6.7–8.8
AST (u/L)	135.28	20.1–355	128.35	14–533	0.628	54–135
BUN (g/dL)	13.28	3.48–117	10.56	3.4–35.71	0.172	7–19
Cre (g/dL)	2.58	1.52–11.2	2.35	1.54–3.65	0.16	0.4–0.9
CK (u/L)	323.47	14.8–5530	254.55	12.4–3800	0.598	88–292
GGT (u/L)	28.33	2.22–235	23.49	9.92–58.1	0.249	17–54

Min=Minimum value, Max=Maximum value,

* Statistically significant at p < 0.05.

a [15]. RBC=Red blood cell, WBC=White blood cell, Hb=Hemoglobin, PCV=Packed cell volume, MCV=Mean corpuscular volume, MCH=Mean corpuscular hemoglobin, MCHC=Mean corpuscular hemoglobin concentration, RDW=Red cell distribution width, PLT=Platelet, PP=Plasma protein, AST=Aspartate aminotransferase, BUN=Blood urea nitrogen, Cre=Creatinine, CK=Creatine kinase, GGT=Gamma-glutamyltransferase

## Discussion

Bullfighting is popular in South Thailand and many people attend the competitions held every weekend [16]. It is a non-lethal traditional sport between bulls and cows. It is also known as bull wrestling or cow fighting [17–20]. Male cattle aged 3–4 years are selected, trained, and prepared for bullfighting. In general, one bull per house is raised and the animals are treated kindly and well cared for. The bulls are primarily raised in South Thailand as draft animals for agricultural activity, meat consumption, and traditional bullfighting [21]. The weather and ecology in South Thailand are suitable for blood-sucking insects because of its hot and humid conditions, and it has widespread areas of vegetation, such as para-rubber plantations, fruit farms, and rice fields. The main vector transmitting theileriosis is *Rhipicephalus microplus*, which is a cattle tick that is distributed in South Thailand [22]. Several reports have indicated the incidence of theileriosis in beef and dairy cattle in four regions of Thailand. However, little information on blood parasite infection in bullfighting cattle is available in the literature. Based on microscopic examination, *Theileria* infection in the study of bullfighting cattle ranged from 1.2% to 38% [12, 23, 24]. The present study is the first to involve molecular detection of *Theileria* spp. infection in bullfighting cattle using *MPSP*-specific genes. *Theileria* *orientalis* was the predominant species among bullfighting cattle. *Theileria orientalis* was also the predominant species (30.1%) infecting beef cattle in North and Northeast Thailand [6]. However, based on the previous reports, the prevalence of *T. orientalis* infection in bullfighting cattle was higher than that of *T. orientalis* in beef and dairy cattle.

The present study evaluated hematological alterations in *Theileria* spp. infected bulls, with a significantly lower RBC level (p < 0.05) compared with *Theileria* spp. uninfected group. This study was similar to the study of Patial *et al*. [25]. *Theileria* *orientalis* infection is primarily associated with RBCs and bovine anemia in cattle, which causes “oriental theileriosis.” This disease affects only the hemoglobin level of cattle in Bali, Indonesia [26]. In India, the clinical manifestations o*f T. orientali*s infection in Holstein–Friesian cattle can cause abortion, arthritis, and severe anemia, but reporting is rare [25]. In the present study, the bulls infected with *T. orientalis* had mild clinical signs of anemia. In addition, some blood biochemical parameters (AST, CK, and Cre) of *Theileria* spp. infected bulls were higher than the reference values. A high AST value may indicate liver damage, whereas a high CK level indicates skeletal muscle injury. Moreover, a high Cre level indicates poor kidney function. These results may be due to exhaustion following bullfighting competitions.

## Conclusion

This study was the first molecular confirmation of *Theileria* spp. infection in bullfighting cattle. *Theileria orientalis* was the dominant infection among bulls. Thus, tick control measures should be promoted to the owners to improve the protective capability of cattle from this parasitic blood protozoan.

## Authors’ Contributions

PR: Carried out study design, sample and data collection, diagnosis, and drafted the manuscript. NK: Performed sampling, data collection, and diagnosis. RN and KK: Analyzed the data and drafted, reviewed and revised the manuscript. All authors have read and approved the final manuscript.

## References

[1] World Organization for Animal Health (2020)-Online World Animal Health Database.

[2] Kakuda T, Kubota S, Sugimoto C, Baek B.K, Yin H, Onuma M (1998). Analysis of immunodominant piroplasm surface protein genes of benign *Theileria* parasites distributed in China and Korea by allele-specific polymerase chain reaction. J. Vet. Med. Sci.

[3] Chansiri K, Kawazu S, Kamio T, Terada Y, Fujisaki K, Philipe H, Sarataphan N (1999). Molecular phylogenetic studies on *Theileria* parasites based on small subunit ribosomal RNA gene sequences. Vet Parasitol.

[4] Sarataphan N, Nilwarangkoon S, Tananyutthawongese C, Kakuda T, Onuma M, Chansiri K (1999). Genetic diversity of major piroplasm surface protein genes and their allelic variants of *Theileria* parasites in Thai cattle. J. Vet. Med. Sci.

[5] Sarataphan N, Kakuda T, Chansiri K, Onuma M (2003). Survey of benign *Theileria* parasites of cattle and buffaloes in Thailand using allele-specific polymerase chain reaction of major piroplasm surface protein gene. J. Vet. Med. Sci.

[6] Jirapattharasate C, Moumouni P.F, Cao S, Iguchi A, Liu M, Wang G, Zhou M, Vudriko P, Changbunjong T, Sungpradit S, Ratanakorn P, Moonarmart W, Sedwisai P, Weluwanarak T, Wongsawang W, Suzuki H, Xuan X (2016). Molecular epidemiology of bovine *Babesia* spp. and *Theileria orientalis* parasites in beef cattle from northern and northeastern Thailand. Parasitol. Int.

[7] Sivakumar T, Tattiyapong M, Fukushi S, Hayashida K, Kothalawala H, Silva SS, Vimalakumar S.C, Kanagaratnam R, Meewewa A.S, Suthaharan K, Puvirajan T, de Silva WK, Igarashi I, Yokoyama N (2014). Genetic characterization of *Babesia* and *Theileria* parasites in water buffaloes in Sri Lanka. Vet. Parasitol.

[8] Jia L, Zhao S, Xie S, Li H, Wang H, Zhang S (2020). Molecular prevalence of *Theileria* infections in cattle in Yanbian, North-eastern China. Parasite.

[9] Kaewhom P, Thitasarn W (2017). The prevalence of *Theileria* spp. of goat in Watthana Nakhon district, Sa Kaeo Province. J. Mahanakorn. Vet. Med.

[10] Chowdhury A (2017). Theileriosis as a Course of Significant Mortality in Pregnant Cattle.

[11] Perera P.K, Gasser R.B, Firestone S.M, Anderson G.A, Malmo J, Davis G, Beggs D.S, Jabbar A (2014). Oriental theileriosis in dairy cows causes a significant milk production loss. Parasit Vectors.

[12] Ngasaman R, Keawchana N, Rakwong P (2021). Haemoparasites infection in bullfighting cattle in southern of Thailand. Vet. Integr. Sci.

[13] Cao S, Zhang S, Jia L, Xue S, Yu L, Kamyingkird K, Moumouni P.F, Moussa A.A, Zhou M, Zhang Y, Terkawi M.A, Masatani T, Nishikawa Y, Xuan X (2013). Molecular detection of *Theileria* species in sheep from Northern China. J. Vet. Med. Sci.

[14] Ota N, Mizuno D, Kuboki N, Igarashi I, Nakamura Y, Yamashina H, Hanzaike T, Fujii K, Onoe S, Hata H, Kondo S, Matsui S, Koga M, Matsumoto K, Inokuma H, Yokoyama N (2009). Epidemiological survey of *Theileria orientalis* infection in grazing cattle in the eastern part of Hokkaido, Japan. J. Vet. Med. Sci.

[15] https://www.vet.cornell.edu/animal-health-diagnostic-center/laboratories/clinical-pathology/reference-intervals/hematology.

[16] Rattana S (2017). Popularity of Bullfighting Grows in South, The Nation Thailand News.

[17] The National Folk Museum of Korea, South Korea (2014). Encyclopedia of Korean Seasonal Customs:Encyclopedia of Korean Folklore and Traditional Culture.

[18] Darke D (2011). Eastern Turkey.

[19] Garrido-Castañé I, Romero A.O, Espuny J.C, Hentrich B, Basso W (2019). *Besnoitia besnoiti* seroprevalence in beef, dairy and bullfighting cattle in Catalonia (North-Eastern Spain):A cross-sectional study. Parasitol. Int.

[20] Kuwahara S, Ozaki T, Nishimura A (2007). Transperipheral networks. Shima Int. J. Res. Isl. Cult.

[21] Vitone J, Boonkaew S (2014). Thai Bullfight-Bullfighting in Southern Thailand. Electronic Book.

[22] Rakthong P, Nooma W, Trinachartvanit W, Ahantarig A (2015). Biodiversity and distribution of ticks in southern Thailand. Rajabhat J. Sci. Humanit. Soc. Sci.

[23] Chethanon U, Srinunthapanth S, Sungvilai C (1987). *Theileria* spp. in Cattle in the South of Thailand I. Bovine *Theileriosis*:A Case Report.

[24] Saengsoi W, Chooruang N (2019). Prevalence of Blood Parasites in Fighting Bulls. Research Summary.

[25] Patial V, Gupta T, Angaria S, Bali D, Katoch A, Gautam M, Singh NK, Sharma M, Chahota R (2021). *Theileria orientalis* outbreak in an organized cattle breeding farm. Vet. Parasitol. Reg. Stud. Rep.

[26] Aziz N, Maksudi M, Prakoso Y.A (2019). Correlation between hematological profile and theileriosis in Bali cattle from Muara Bulian, Jambi, Indonesia. Vet, World.

